# Characterization of mind wandering using fNIRS

**DOI:** 10.3389/fnsys.2015.00045

**Published:** 2015-03-26

**Authors:** Gautier Durantin, Frederic Dehais, Arnaud Delorme

**Affiliations:** ^1^Département Conception des Véhicules Aérospatiaux, Institut Supérieur de l’Aéronautique et de l’EspaceToulouse, France; ^2^Centre de Recherche Cerveau et Cognition, Universite de ToulouseUPS, Toulouse, France; ^3^CNRS, CerCoToulouse, France

**Keywords:** mind wandering, fNIRS, linear discriminant analysis, default mode network

## Abstract

Assessing whether someone is attending to a task has become important for educational and professional applications. Such attentional drifts are usually termed mind wandering (MW). The purpose of the current study is to test to what extent a recent neural imaging modality can be used to detect MW episodes. Functional near infrared spectroscopy is a non-invasive neuroimaging technique that has never been used so far to measure MW. We used the Sustained Attention to Response Task (SART) to assess when subjects attention leaves a primary task. Sixteen-channel fNIRS data were collected over frontal cortices. We observed significant activations over the medial prefrontal cortex (mPFC) during MW, a brain region associated with the default mode network (DMN). fNIRS data were used to classify MW data above chance level. In line with previous brain-imaging studies, our results confirm the ability of fNIRS to detect Default Network activations in the context of MW.

## Introduction

While reading books, people’s attention may drift towards self-centered matters. After some time, the readers may realize that they have lost track of their reading and have started to mind wander. These attentional drifts are called mind wandering (MW) episodes and people are generally unaware of when they occur. Avoiding these attentional drifts is not only a matter of will power since even after years of practice focused meditation, meditators still experience these drifts regardless of their efforts to avoid them (Braboszcz et al., 2010). The assessment of MW events may be of importance for several potential applications. Since attentional deficit/hyperactivity disorder (ADHD) patients also have higher occurrence of MW episodes (Peterson et al., 2009), determining the amount of MW would be beneficial for those individuals. This could also have benefits for the science of education since MW may prevent the assimilation of educational material (Szpunar et al., 2013). Finally, as MW reduces the cortical processing of the external environment (Mooneyham and Schooler, 2013), it may jeopardize safety in operational situations such as driving a vehicle (He et al., 2011; Galéra et al., 2012) or flying a plane (Casner and Schooler, 2014).

Studies on MW have usually involved sustained attention paradigms such as breath counting (Braboszcz et al., 2010) or go-no go tasks (Shaw et al., 2013). One example of a go-no go task includes the popular Sustained Attention to Response Task (SART), wherein single digit numbers appear one at a time on a computer screen: subjects are instructed to press a button whenever a number other than the target number (3) appears (Manly et al., 1999). The SART is simple so subjects’ attention frequently leaves the primary task, in addition to being sensitive to the tendency for participants to automate their behavior. During episodes of MW, subjects tend to press the button systematically (even when three appears). By counting the number of successive missed targets, it is possible to assess the time and frequency of MW episodes. As outlined in Smallwood and Schooler (Smallwood and Schooler, 2006), investigations of the experience of MW during the SART support that, first, blocks in which this phenomenon occurs are associated with faster response times than are blocks in which attention is directed towards the task. Second, high levels of MW reported with retrospective questionnaires are associated with a tendency to make an error during periods of task disengagement. We will primarily focus on the second point—errors during the SART—to process fNIRS data.

Due to its importance, studies aiming at characterizing MW using various objective psychophysiological measurements have flourished in the last years (Smallwood and Andrews-Hanna, 2013). For instance, findings from a study (Smallwood et al., 2008) in which participants received experience sampling probes while performing the SART showed that the amplitude of a late positive ERP component of the event related potentials measured by EEG (known as P3) was reduced by MW, confirming the hypothesis that MW induces decoupling from the environment. Christoff et al. (2009) investigated functional Magnetic Resonance Imaging (fMRI) samples while participants were engaged in the SART task. Interestingly, they found evidence of activation of Default Mode Network (DMN)—known to exert high activity levels during off-task conditions—and recruitment of executive network regions during MW episodes. Numerous other studies using sustained attention tasks, showed a greater implication of the right hemisphere in the process of sustained attention (Warm et al., 2009; Stevenson et al., 2011).

However, the importance of MW phenomenon both in laboratory experiments and in daily life (Killingsworth and Gilbert, 2010; Mooneyham and Schooler, 2013) calls for means to characterize further its neurophysiological correlates in real-life conditions using portable solutions.

Functional near infra-red spectroscopy (fNIRS) is a non-invasive neuroimaging technique that is portable, relatively low-cost and has high spatial resolution, which makes it a promising technique for research (Strait and Scheutz, 2014). fNIRS has successfully been used for the monitoring of attentional states (Harrivel et al., 2013), vigilance (Warm et al., 2009; Helton et al., 2013) and task unrelated thoughts (Stevenson et al). In this study, we used the fNIRS in a SART task as a mean to characterize MW with a classification approach. The objective of this experiment is to assess fNIRS sensitivity to measure neural correlates of MW, and to discriminate single trial MW vs. non-MW episodes using formal classification.

## Material and Methods

### Participants

The experiment was approved by local ethics committee (Comité de Protection des Personnes). Twenty-three male students from ISAE school of engineering gave their consent to participate in the experiment (21 right-handed; age range 21–24 years, mean age: 22.6). All participants reported normal or corrected vision, and none were suffering from neuropsychological problems.

Prior to the experiment, subjects were provided instructions for the SART task. No explanations were given about the phenomenon of MW, in order to avoid this having an impact on their performance.

### SART

**Figure 1:**
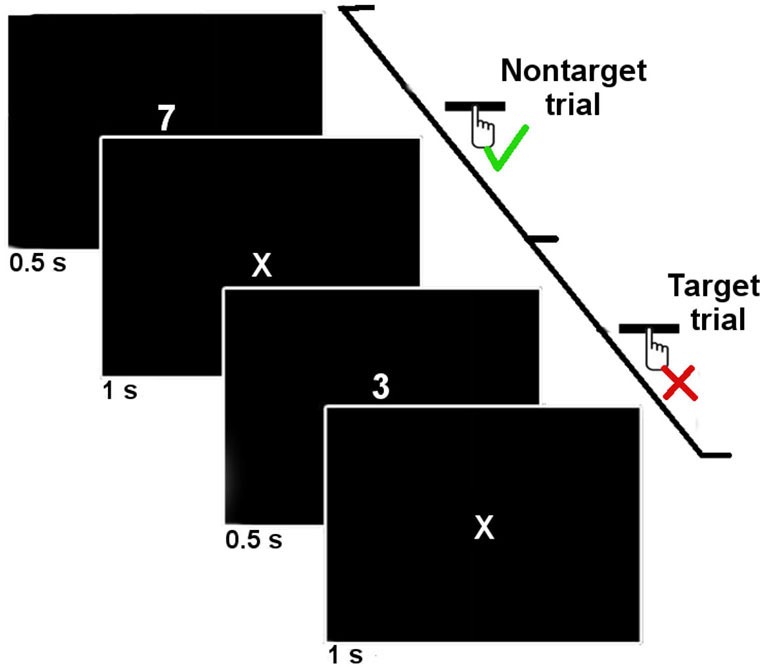
**Time course of two trials of the SART protocol**. The digits were presented 500 ms with an onset-to-onset interval of 1500 ms. Subjects were asked to press the spacebar when a non-target digit was presented, and not to respond when a target digit (3) was presented.

Subjects were asked to perform a computerized SART task. The SART task consists of a simple go/no-go task in which a single infrequent target digit is presented (here the digit 3) amongst frequent non-targets digits (1–9). The computer screen was placed approximately 70 cm from the participants’ head. Each digit was presented for 500 ms on the computer screen and then replaced by a fixation mark (“*X*”) for 1000 ms (see Figure 1). Digits appeared in white on a black background and were approximately 3 cm high in Arial font. The participants were asked to press the spacebar of the computer keyboard for non-target digits, and not to press it if the digit was a target (3). The target trials for which the participants inaccurately pressed the spacebar were considered “SART Errors”. In the rest of this manuscript, we will designate by the term “SART No errors” the other target trials.

The experiment consisted of the presentation of two blocks of 198 trials, 22 of them (11%) being target trials. Targets presentation was pseudorandom, and ensured that two target trials would not follow each other. The mean interval between two target trials was 11.6 s (SE: 3.35 s). Preliminary to the experiment, for training purposes, subjects performed 18 practice trials (2 of which were targets). Subjects were told to respond as accurately and as quickly as possible to digit presentations.

At the end of the experiment, subjects were asked to complete a questionnaire where they could report their opinion about the task and whether they experienced any difficulties. In the questionnaire, we also asked subjects whether they had any thoughts that were unrelated to the task (Gruberger et al., 2011).

### Data Acquisition

**Figure 2:**
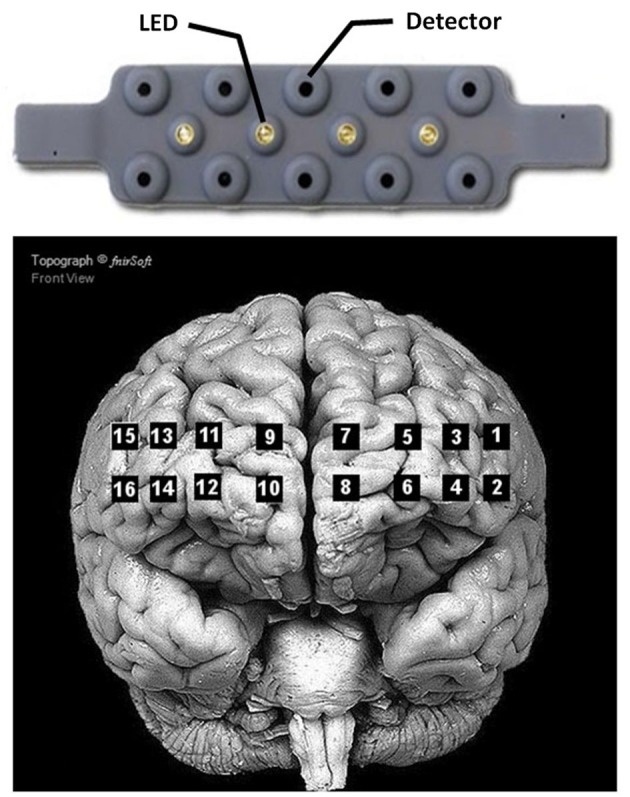
**Arrangement of the 4 LED sources and 10 detectors of the Biopac^®^ fNIR100 device (top), and location of the corresponding optodes on the cortex (bottom)**. Adapted from fnirSoft® software for NIRS data analyses.

During the experiment, hemodynamic data from the prefrontal cortex were recorded using a fNIR100 device (Biopac Inc.). The device consists of four light-emitting diodes (LED) sources of 730 nm and 850 nm (LED current: 12 mA), and ten detectors (see Figure 2 for arrangement). The source and detectors are separated by 2.5 cm, resulting in 16 optodes uniformly placed on a rectangular grid on the forehead (see Figure 2). Data was collected with a sampling frequency of 2 Hz. A baseline of 10 s at the beginning of the experiment was used to calibrate the device. Optodes 1, 3 and 5 were defective and removed from all subjects.

### Data Processing

The 95% confidence intervals for the number of SART errors committed during the task were calculated using the SingleBayes method from Crawford and Garthwaite (Crawford and Garthwaite, 2007), and the probability density function of the apparition of SART errors during the experiment was estimated using Statistica®, using periods of 30 s and Weibull model estimation, a probability distribution commonly used in survival analysis for event density probability function estimation (Kennedy and Gehan, 1971).

The density function is defined as follow:

(1) $$pdf\left(t\right)=\frac{P\left(t<SART\;Error\;<t+dt\right)}{dt}$$

Hemodynamic data recorded by fNIRS were processed using the EEGLAB toolbox for MatLab® (Delorme and Makeig, 2004). Continuous data were high-pass filtered using a short non-linear Infinite Impulse Response (IIR) filter of order 6 and a cutoff frequency of 0.02 Hz. We extracted epochs from continuous data for the SART Error and SART No Error conditions, starting 30 s before stimulus apparition, and stopping 10 s after. The significance of the hemodynamic changes mapped using fNIRS was tested at the topographical level, using the Montecarlo Statistics and using the Cluster Correction for multiple comparisons (Maris and Oostenveld, 2007) to correct for multiple optodes measurements.

### Classification

We used Linear Discriminant Analysis (LDA) for the classification of MW epochs as compared to epochs in which subjects were concentrated. Non-target epochs were not considered here. We considered only MW epochs, with either a response (correct detection) or no response (incorrect detection). LDA is a basic classifier that is robust and fast to compute. We used a 10-fold cross-validation approach to test our model. This means that there are 10 iterations to the algorithm. For each iteration, 90% of the data is used for learning the LDA weights and the remaining 10% of the data is used for testing the model the data for testing is always different from the previous iterations.

## Results

### Behavioral Results

**Figure 3:**
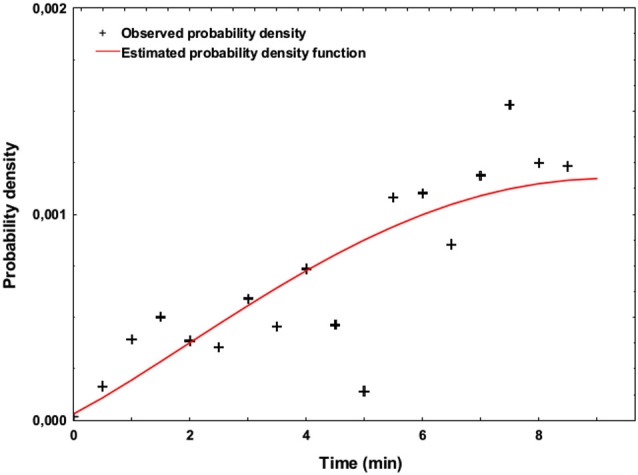
**Estimated probability density function representing the occurrence of SART errors across time (Weibull model, estimated over the two blocks of the experiment using unweigthed least-squares regression)**.

Over the 44 target trials of the SART task, the participants made a mean of 12.7 errors (standard deviation: 7.0), which represents 29% of the target trials. Figure 3 shows the probability density function of the apparition of SART errors during the two sessions of the experiment. This figure exhibits the increasing SART Errors density over time.

At the end of the experiment, 19 out of the 23 participants stated in the questionnaires that they found it difficult to stay focused on the task and had irrelevant thoughts during the experiment. The four subjects who did not mention this fact committed an average of 10.8 errors (SD: 6.45), whereas the 19 subjects conscious of MW committed on average 13.1 errors (SD: 7.37). Although the small sample size for the first group of four subjects did not permit group level statistical testing, the individual comparison of each of these subjects with the group of subjects who reported MW revealed that none of the subjects who were not aware of MW committed significantly less SART Errors than the 19 others.

### Hemodynamics

**Figure 4:**
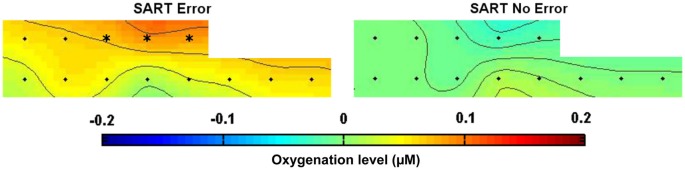
**Topography of HbO_2_ concentration over the prefrontal cortex during SART Error (on the left) and SART No Error (on the right) conditions, averaged [−15 s; −5 s] before the apparition of the target stimulus across all subjects**. The color code represents the level of HbO_2_ concentration changes relative to baseline (in µM). Optodes exhibiting significant differences (all in the mPFC) are marked with a * (significance level = 0.01 after correction for multiple comparisons).

**Figure 5:**
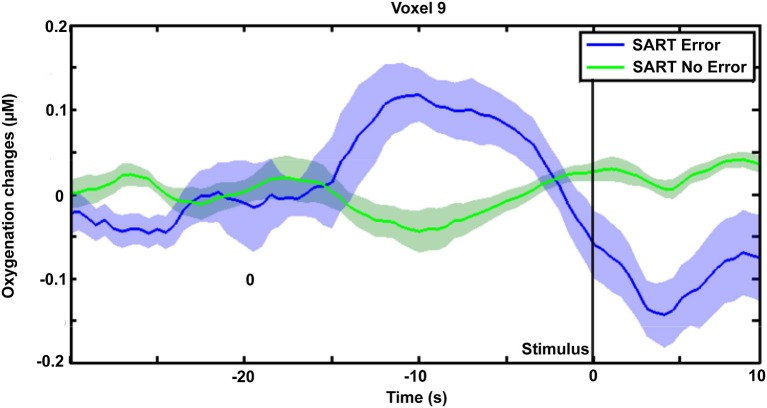
**Variation of HbO_2_ concentration (averaged trials across subjects) on optode 9 (in the mPFC) preceding SART Error (in blue) and SART No Error trials (in green)**. Shaded areas represent the standard error of the mean for each condition.

Figure 4 shows the topography of HbO_2_ concentration under both SART Error and SART No Error conditions, from 15 s until 5 s before the apparition of the stimulus. After correcting for multiple comparisons, our analysis revealed significantly higher levels of oxygenated hemoglobin for optodes 7, 9 and 11, located in the dorsomedial prefrontal cortex, preceding SART Errors (associated with MW episodes). Figure 5 indicates the temporal dynamics associated with optode 9, and shows that the greater activation observed in the medial prefrontal cortex (mPFC) before SART Errors returns to normal before the arrival of the stimulus. No significant variations relative to SART Errors were found on the deoxy-hemoglobin (HHb) signal.

### Classification

**Figure 6:**
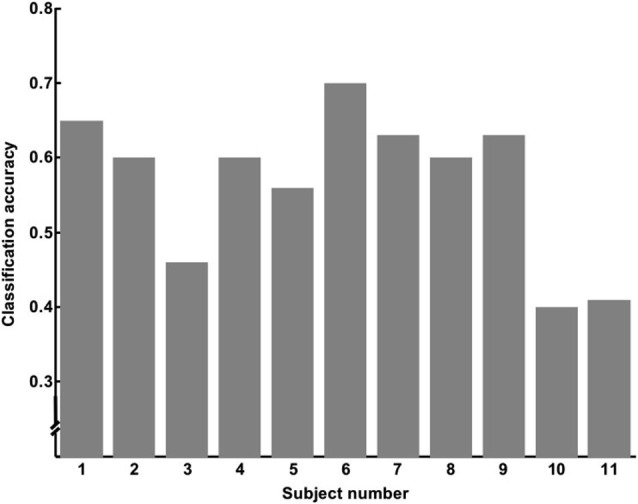
**Accuracy per subject obtained for classification of SART Error vs. SART No Error trials**.

We used data from only 11 subjects to classify SART Error and SART No Error trials, keeping only the subjects who made at least 10 errors, so we would have enough trials to train and test the classifier. Out of the 11 subjects, 7 of them had a classification accuracy superior to 60%. A Wilcoxon sign test showed that this result was unlikely to occur by chance (*p* < 0.016; degree of freedom of 10). Results are summarized in Table 1, and Figure 6 shows the individual accuracy results.

**Table 1:** **Mean mind wandering episodes classification performance across subjects**.

	Mean	Standard error
**Accuracy**	56%	2.70%
**Sensitivity**	52%	6.33%
**Specificity**	62%	4.82%

## Discussion

We showed that MW occurred during the SART, as 19 out of 23 participants retrospectively reported MW meta-awareness during the experiment (Smallwood and Schooler, 2006). The high number of errors made during target trials is consistent with SART characteristics regarding MW (Manly et al., 1999), and notably the increasing occurrence of MW episodes with the time spent on the task as shown in Figure 3, are consistent with previous findings using sustained attention tasks (Mackworth, 1948; Allan Cheyne et al., 2009). However, contrary to Smallwood and Schooler’s study, the retrospective awareness of MW could not be associated with a higher number of SART errors. Although 4 out of 23 subjects did not report experiencing MW, it is noticeable this was not associated with the absence of SART Errors (mean number of errors: 10.8), therefore showing that SART Errors occurred during the experiment, even without MW meta-awareness.

Our results showed that it is possible to discriminate responses where subjects are not paying attention to the task, compared to periods where they are attending based solely on the fNIRS signal. We observed significant differences in the fNIRS signal measured in the mPFC preceding the cue presentation for correct vs. incorrect trials. Notably, the medial prefrontal activations we observed prior to SART errors (see Figure 4) were similar to activations observed previously in fMRI with the same task (Christoff et al., 2009). These results, in conjunction with previous neuroimaging MW investigations (Mason et al., 2007), support the feasibility of using fNIRS to detect DMN activity (Sasai et al., 2012), and its implication in the phenomenon of MW. Our results did not show implication of the Executive Network areas such as dorsolateral prefrontal cortex (DLPFC) during SART errors, as revealed by two previous fMRI studies (Christoff et al., 2009; Stawarczyk et al., 2011). In these studies, DLPFC activation was related to MW meta-awareness but not to SART Errors. However, our experimental paradigm was not designed to measure MW meta-awareness and thus did not allow to state about DLPFC activations. Moreover, the absence of left DLPFC activations could be due to the fact that the optodes in this area were defective and had to be removed from the study.

An important contribution of our study concerns the temporal dynamics of the activations observed in the mPFC. This area did not show sustained activation during MW episodes, as the level of HbO_2_ measured faded even before target stimulus apparition (see. Figure 5). Similarly, previous investigation of MW using SART protocol in fMRI only found DMN activations preceding MW occurrences (Christoff et al., 2009; Stawarczyk et al., 2011). This would suggest that the contribution of the mPFC to the DMN is the strongest at the beginning of the MW episode, which would be consistent with the role of mPFC during sleep as shown by functional connectivity MRI studies (Sämann et al., 2011). Hence, this transient activation of the mPFC could suggest that this brain area plays a role to switch from a concentrated to a MW state. However, further investigation using self-caught MW protocols and analyzing the temporal dynamics of brain activation would be needed to support this hypothesis.

Some limitations remain concerning the interpretation of SART Errors. Although previous research has demonstrated that performance on the SART task is mainly determined by the capacity to endogenously sustain attention (Manly et al., 1999), there remains an ongoing debate as to whether SART errors could be related to impulsivity in subjects’ responses (Helton et al., 2009; Stevenson et al., 2011). Nevertheless, previous investigations including both SART and experience sampling experiments performed online support that SART errors are linked to MW (Christoff et al., 2009). In addition, one may argue that our results on SART errors trials could correspond to the absence of motor response inhibition. Despite the high rate of retrospective assessment of MW, the occurrence of SART Errors even without meta-awareness during the experiment suggests that MW alone cannot account for the totality of the SART Errors. Nevertheless, further investigation is needed concerning the presence or absence of left DLPFC activations, as the voxels in this area had to be removed, and to confirm that the activations measured relate only to MW.

Classification accuracy showed that single-trial classification returned relatively poor results, although significantly better than chance, indicating that real time detection of such events using only fNIRS signal would be difficult to achieve. These results could in part be due to the high inter-subject variability observed with fNIRS signals (Jasdzewski et al., 2003; Sato et al., 2005). Moreover, the temporal closeness between target trials, due to their rate among all the trials (11%) may have potentially jeopardized single-trial classification. Although further investigation using a modified protocol is needed to eliminate the potential confound, the low classification accuracy we obtained does not make fNIRS a good candidate to detect MW in real time, when used alone. Nevertheless, the significance of the classification compared to chance suggests that the fNIRS signal could complement other methodologies such as pupil diameter (Grandchamp et al., 2014) or electro-encephalography (Braboszcz et al., 2010) to improve classification performance, and predict MW before subjects become aware of them (O’Connell et al., 2009).

## Conflict of Interest Statement

The authors declare that the research was conducted in the absence of any commercial or financial relationships that could be construed as a potential conflict of interest.
